# The prevalence and pattern of HPV-16 immunostaining in uterine cervical carcinomas in Ethiopian women: a pilot study

**DOI:** 10.4314/pamj.v8i1.71077

**Published:** 2011-03-11

**Authors:** Mona M Rashed, Alemayehu Bekele

**Affiliations:** 1 Department of Pathology, Affiliated to General Organization of Teaching Hospitals and Institutes, Egypt; 2 Department of Pathology, Affiliated to Jimma University, Ethiopia

**Keywords:** Cancer cervix, HPV 16, immunohistochemistry, Ethiopia

## Abstract

**Introduction:**

Cancer of the cervix uteri is the second most common cancer among women worldwide. The association of human papillomavirus (HPV) infection with cervical carcinogenesis is well documented. This is a pilot study aiming to studying the prevalence and the pattern of Human Papilloma Virus Type 16 (HPV16) by immunostaining in the tissues of cervical carcinomas of Ethiopian women.

**Methods:**

20 specimens of uterine cervical carcinomas were studied histopathologically and immunohistochemically for HPV16.

**Results:**

Histologically the specimens were classified as: Ten cases were Non Keratinized Squamous cell carcinoma (NKSCC), six cases were Keratinized Squamous Cell Carcinoma (KSCC) and four cases were Adenocarcinoma (ADC). Immunohistochemistry study showed positivity in eleven cases (55%); seven cases (35%) were non-keratinized squamous cell carcinoma; three cases (15%) were keratinized squamous cell carcinoma and one case (5%) belonged to the adenocarcinomas.

**Conclusion:**

This study reveals a significant detection of HPV in Ethiopian women by the use of advanced techniques such as Immunohistochemistry (IHC). The data of this study suggested that the marked expression of the HPV 16 was in the less differentiated uterine cervix carcinomas.

## Introduction

Cervical cancer is an important public health problem for adult women in developing countries in South and Central America, sub-Saharan Africa, and south and south-east Asia [1,2]. The International Agency for Research on Cancer (IARC) of the World Health Organization (WHO) estimated that nearly 80 percent of cervical cancer cases occur in developing countries and, in many such regions, it is the most common cancer and cause of death from cancer among women [3]. Within Africa, the incidence and prevalence estimates vary widely between geographical regions. Five of the seven regions with the highest incidence rates in the world are in eastern and Sub-Saharan (southern) Africa, while in northern Africa the incidence is lower [3,4]. 

Retrospective analysis of the causes of postmenopausal bleeding in Addis Ababa, Ethiopia; showed that cervical carcinoma accounted for 84.8% of all female genital tract malignancies [5]. Cervical carcinoma was most common malignant disease in females in retrospective study of malignant neoplasm at Yirga Alem Hospital in Southern Ethiopia [6]. According to the Publications of the WHO/ICO Information Centre on HPV and Cervical Cancer5 in Ethiopia, every year 4648 women are diagnosed with cervical cancer and 3235 die from the disease. Cervical cancer ranks as the 2nd most frequent cancer among women in Ethiopia. Data is not yet available on the HPV burden in the general population of Ethiopia. However, in Eastern Africa, the region Ethiopia belongs to, about 33.6% of women in the general population are estimated to harbor cervical HPV infection at a given time [7]. There is no published epidemiologic or clinical study on cervical carcinoma here in South-Western part of Ethiopia, yet cervical carcinoma found to be most common malignant neoplasm in unpublished data from Department of Medical Laboratory Sciences and Pathology Jimma University. 

The vast majority of cervical cancer cases are caused by infection with certain subtypes of human papilloma virus (HPV), a sexually transmitted virus that infects cells and may result in precancerous lesions and invasive cancer [8,9]. The highest regional prevalence of HPV infection was found to be in Africa, where 22% of women had evidence of HPV infection while the world wide prevalence of HPV is 10.4 % [10]. Women are generally infected with HPV in their teens, 20s, or 30s; it can take as long as 20 years after HPV infection for the cancer to develop. The prevalence of cervical HPV infection decreases sharply in women after the age of 30 [11]. The American Cancer Society provides the following list of risk factors for cervical cancer: Human Papillomavirus (HPV) infection, smoking, HIV infection, chlamydia infection, dietary factors, hormonal contraception, multiple pregnancies, exposure to the hormonal drug diethylstilbestrol (DES) and a family history of cervical cancer. These factors probably modify the risk in women infected with HPV [12]. 

The present study is a retrospective pilot study aiming to determining the prevalence of Human Papilloma Virus Type 16 (HPV16) associated histological types of cervical carcinomas in Ethiopian women. 

## Methods

This pilot feasibility study was designed prior to a larger immunohistochemistry studies related to cancer cervix and to improve the efficacy and quality of immunostaining procedures in the Department of Medical Laboratory Sciences and Pathology; College of Public Health and Medical Sciences, Jimma University. 

The study included 20 punch biopsy specimens of uterine cervical carcinomas retrieved randomly from the archives of the Department of Medical Laboratory Sciences and Pathology; Collage of Public Health and Medical Sciences; Jimma University. All patients were presented clinically with irregular vaginal bleeding or post coital bleeding. The retrieved punch biopsies were sectioned and stained by hematoxylin and eosin stain for histopathological classification of cases according to the (IARC/WHO); World Health Organization (WHO) classification of cervical tumors [13]. 

Immunohistochemistry was performed on the 20 cervical carcinomas specimens. Four micrometer sections of formalin-fixed, paraffin-embedded tissues were cut and placed on coated slides. The sections were dewaxed in xylene, rehydrated in graded alcohol, and rinsed in water. For antigen retrieval, the sections were immersed in 0.01M of citrate buffer, pH 6.0, in a high pressure cooker for 10 minutes, the tissue sections were cooled under tap water for 10 minutes. A peroxidase block reagent was applied and it was incubated for 5-10 minutes at room temperature. Next, a block reagent was added, after draining out the slides, and incubated for 15 minutes at room temperature. The monoclonal antibody (HPV16; BioGenex Laboratories, Inc. USA) was added for one hour on the specimen followed by the secondary antibody (DAKO, Copenhagen, Denmark) that was incubated for 30 minutes, followed by rinsing with Tris buffer saline (pH 7.4 to 7.6) for 10 minutes. The slides were drained and blotted around the sections, to which an appropriate volume of substrate (3, 3"-diaminobenzdine) solution was added, and they were incubated for 40-50 minutes at room temperature followed by rinsing in Tris-buffer saline. Finally, the sections were counterstained in a Mayer"s hematoxylin bath for 5 minutes and then rinsed with tap water. Known positive control sections were included in each run to ensure proper immunostaining whereas the negative control consisted of the same section where the primary antibody was omitted. 

## Results

The pilot study included 20 cases of cancer cervix; the patient ages ranged from 32 years to 56 years, the clinical presentations were mostly vaginal bleeding for more than one month. Histologically the specimens were classified as: Ten cases (50%) were Non Keratinized Squamous cell carcinoma (NKSCC), six cases (30%) were Keratinized Squamous Cell Carcinoma (KSCC) and four cases (20%) were Adenocarcinoma (ADC).

**Figure 1:**
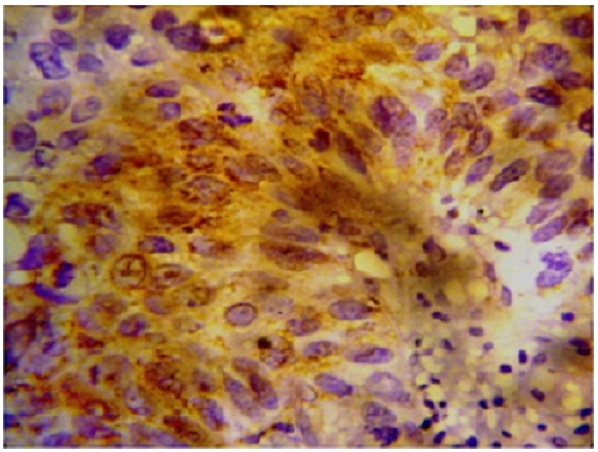
HPV16 immonostaining was seen granular, cytoplasmic, perinuclear and nuclear in a non-keratinized squamous cell carcinoma (NKSCC) uterine cancer cervix (x400)

**Figure 2:**
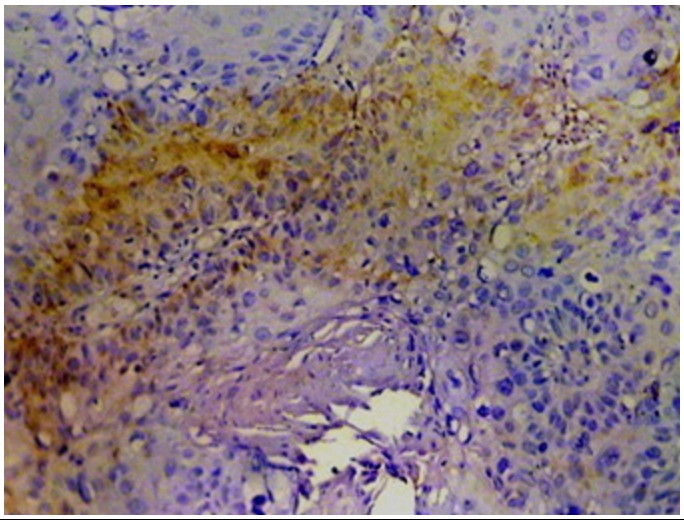
HPV positive immunostaining in keratinized squamous cell carcinoma (KSCC) of uterine cancer cervix (x200)

**Figure 3:**
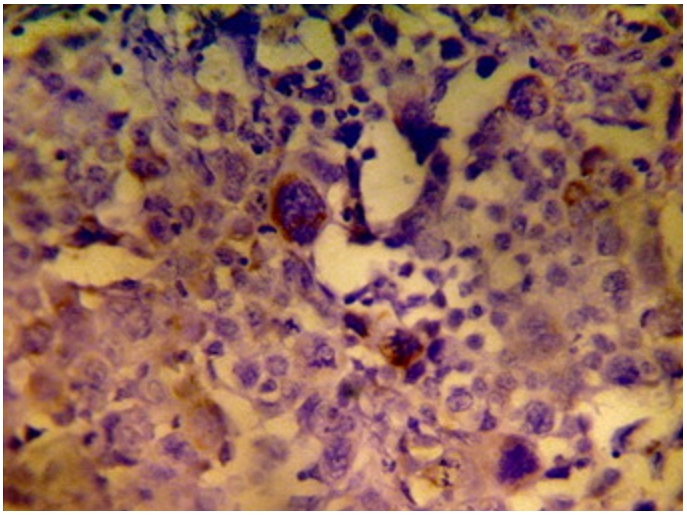
Adenocarcinoma grade III of uterine cancer cervix; the HPV16 was focally positive, even positivity was related to single cells (x400)

**Figure 4:**
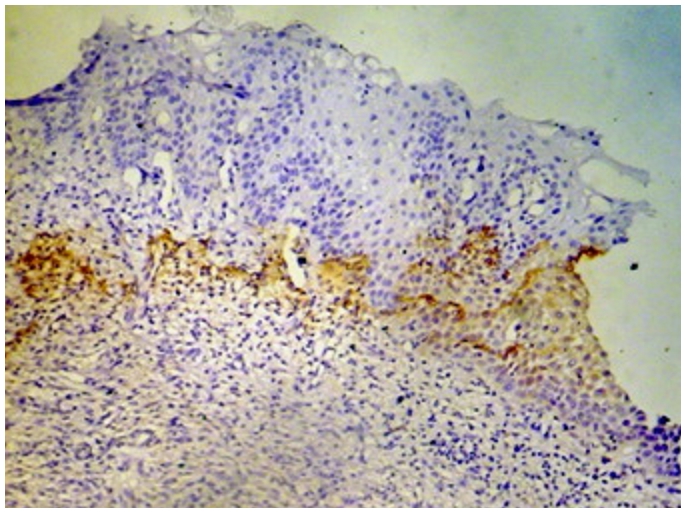
Squamous dysplasia in uterine cervix, the HPV16 positive immunostaining was mainly in the basal and the parabasal epithelial cells (x100)

Immunohistochemistry study showed positivity in eleven cases (55%); the staining reaction was seen granular, cytoplasmic and also perinuclear and nuclear. The immunostaining positive reactions were distributed as; seven cases (35%) non-keratinized squamous cell carcinoma; three cases (15%) were keratinized squamous cell carcinoma and one case (5%) belonged to the adenocarcinomas. In the sheets of tumor cells, HPV was positive in the less mature cells (Figure 1); meanwhile most of the mature keratinized squamous cells were mostly weak positive or negative, only in one focus HPV was positive in a keratinizing squamous pearl (Figure 2). In one case, that was adenocarcinoma grade III; the HPV16 was focally positive, even positivity was related to single cells (Figure 3). The accompanied squamous dysplasia in the studied sections also showed positive immunoreactivity for the HPV16; it was seen positive mainly in the basal and the parabasal epithelial cells, specially those with kilocytic changes; HPV was evidently positive in the areas with cervical epithelial dysplasia (Figure 4). 

## Discussion

The association of Human Papillomavirus (HPV) infection with cervical carcinogenesis is well documented [14]. Nearly all cervical cancers are directly linked to previous infection with one or more of the oncogenic types of HPV [15]. The link between genital HPV infections and cervical cancer was first demonstrated in the early 1980s by Harold zur Hausen, a German virologist. Since then, the link between HPV and cervical squamous cell carcinoma has become well established [16]. 

Studies have shown that >99% of invasive cervical cancers contain high-risk HPV-DNA and HPV-16 is the most prevalent type with an incidence of ~53% [15]. In the present study the prevalence of the HPV16 was 55%. According to different studies, immunohistochemistry (IHC) has a sensitivity of 52-87% for the detection of HPV [17]. 

A prevalence of 15.9% was revealed by study done to determine the prevalence of HPV infection among women attending outpatients' clinics in Attat Rural Hospital Southern Ethiopia [18]. A retrospective molecular analysis for Human Papillomavirus on 284 formalin-fixed and paraffin-embedded cervical biopsy specimens with cervical abnormalities from the Department of pathology, Gondar College of Medical Sciences, Gondar, Ethiopia at Max-Burger Research Institute, Leipzig, Germany revealed HPV prevalence of 92.6 % in that population. Human Papilloma Virus type 16 was identified to be the most frequent genotype accounting for more than 76% of all HPV species in this study [19]. 

An Indian study reported that out of thirty samples, 15 expressed positive and 15 negative for HPV marker [20]. The prevalence in others studies were variable; and this variability may be contributed to ethnic and geographic factors. The prevalence rate of HPV 16 among Iranian patients [21] with cervical carcinoma was reported to be 6.7%. In the present study, the prevalence of HPV 16 among patients with cervical cancer was higher [22] than Croatia (50%), Australia (53%), Thailand (41%), Italy (32.6%), China (48.8%), and the Philippines (43.9%); and was lower than in Colombia (69.9%), Spain (66.4%), and Morocco (72.4%) [20]. 

Squamous cell carcinoma is the predominant type of cancer of the uterine cervix, and HPV 16 is the most common type of HPV DNA in these tumors [14]. Infection with Human Papillomavirus (HPV) is considered to be the principal causal agent in the development of squamous cell carcinoma of the uterine cervix [14,23]. The squamous cell carcinoma represented the majority in the present study (16 cases; 80%) and they were classified histologically as Keratinized types (6 cases; 30%) and this group represent the well differentiated squamous cell carcinoma while the remaining were non keratinized squamous cell carcinoma (10 cases; 50%) represented the less differentiated group of squamous cell carcinoma of uterine cervix. The degree of keratinization (differentiation) is a pathological feature of cervical tumors, in this study, the intensity of HPV immunostain expression was inversely correlated with the differentiation grade of the tumor area, so that the less differentiated areas showed a stronger positive immunostain. 

It is known that during HPV infection, cells in the basal layer are infected first and the production of viral progeny takes place in differentiated cells in intermediate layers [15]. This finding was evident in the present study, the HPV16 positive immunoreactivity in the accompanied squamous dysplasia of the studied sections showed positive HPV16 in the basal and the parabasal epithelial cells, especially those with kilocytic changes. 

Adenocarcinoma, adenosquamous carcinoma, and small-cell carcinoma of the uterine cervix are reported to be low in incidence but clinically important. They usually exhibit a more aggressive biologic behavior and have a poorer prognosis than squamous cell carcinomas at similar stages [24]. Unlike SCC, however, the risk factors for ADC of the cervix are not well understood [14]. The etiopathogenesis of adenocarcinoma is not yet clearly understood. Recent studies have raised more controversy, rather than answering the question of whether specific HPV infection also plays a role in the development of adenocarcinoma of the cervix [24]. There have been several reports showing the presence of HPV DNA, predominantly HPV type 18, in ADC and ADSC in contrast to invasive SCC, in which the incidence of HPV 16 is very high [14]. 

## Conclusion

This study reveals a significant detection of HPV in Ethiopian women by the use of advanced techniques such as Immunohistochemistry (IHC). The data of this study suggested that the marked expression of the HPV 16 was in the less differentiated uterine cervix carcinomas. 

## Competing interests

The authors declare no competing interests. 

## Authors' contributions

Dr MR and Dr AB equally contributed to the study, both are pathologists. Dr. AB supplied the specimens and the data of cases as well as the histopathological interpretation of slides and Dr. MR performed the immunohistochemistry study of the specimens. All authors have read and approved the final version of the manuscript.
